# Research priorities to address supportive care needs in acute myeloid leukaemia (AML)—results from a Delphi survey

**DOI:** 10.1007/s00520-025-09888-7

**Published:** 2025-09-11

**Authors:** Catriona Parker, Robert Weinkove, Andrew H Wei, Zoe K McQuilten

**Affiliations:** 1 Transfusion Research Unit, School of Public Health and Preventive Medicine, Monash University, Melbourne, Australia; 2 Cancer Immunotherapy Programme, Malaghan Institute of Medical Research, Wellington, New Zealand; 3 Wellington Blood and Cancer Centre, Wellington Hospital, Wellington, New Zealand; 4 Peter MacCallum Cancer Centre, Royal Melbourne Hospital, Melbourne, Australia; 5 Walter Eliza Hill Institute of Medical Research and University of Melbourne, Melbourne, Australia; 6 Clinical Haematology, Alfred Health, Melbourne, Australia

**Keywords:** Acute myeloid leukaemia, Supportive care, Prioritisation

## Abstract

**Purpose:**

A cute myeloid leukaemia (AML) carries major clinical, economic, and psychosocial burdens. Supportive care is critical to prevent mortality and morbidity and support long-term well-being. Many AML trials focus on treating AML disease, but few prospectively evaluate supportive care measures. This study sought to identify AML supportive care research priorities to inform the design of future AML clinical trials.

**Methods:**

A three-stage online Delphi process was used. Participants (clinicians and people with lived experience) were invited from Australia and New Zealand through the Australasian Leukaemia and Lymphoma Group (ALLG), the Leukaemia Foundation, and professional networks. Round 1 generated supportive care research question, and in round 2, participants rated each question’s importance. A priori criteria were applied for a question to continue to round three. Finally, participants ranked their top 10 research questions and the type of research needed to address each question.

**Results:**

A total of 31 participants completed round one, and 22 in rounds two and three. People with lived experience made up 32% of the sample in round one, 45% in round two, and 27% in round three. Round one resulted in 66 research questions across 13 domains. In round two, from the initial 66 questions, 16 (24%) met the criteria to progress to the next round. In the final round, participants agreed on 10 priority research questions.

**Conclusion:**

This Delphi process identified ten supportive care research priorities in AML across many domains, including infection management, bleeding prevention, nutrition, exercise, and frailty assessments. This work, which incorporates people with lived experience, can be used to inform design of future AML trials.

**Supplementary Information:**

The online version contains supplementary material available at 10.1007/s00520-025-09888-7.

## Relevance

People with AML have complex and broad supportive care needs often long-lasting into survivorship, but many of these practices are not supported by robust evidence. As a relatively rare disease, progress in generating evidence for supportive care practices for people with AML is exacerbated by low availability of participants. As a research community, we need to work together to answer the most pressing supportive care research questions to improve the quality of life and outcomes of people with AML. This research uses the opinions of subject matter experts including patients to identify the most important supportive care research priorities for AML.

## Background

Acute myeloid leukaemia (AML) is a blood cancer that is often rapidly fatal without treatment. Curative treatment usually consists of intensive induction followed by consolidation chemotherapy and sometimes haematopoietic stem cell transplant to reduce the risk of relapse [1]. For older adults or those with co-morbidities, non-intensive therapies can prolong survival, although are not considered curative. Most patients are transfusion-dependent at some point during treatment, and both short- and long-term complications of treatment are common, requiring a multidisciplinary management approach [2]. Around 25% of people with AML require an intensive care admission for issues such as respiratory failure, septic shock, neurological problems, cardiotoxicity, or bleeding [3].

In an American cross-sectional study of 1182 people affected by AML (901 patients and 281 caregivers), one-third of respondents suffered severe short-term symptoms such as mouth sores, infection, and diarrhoea. Long-term side effects included fatigue reported by 25% of respondents, cognitive dysfunction (12%), neuropathy (10%), and severe organ damage (9%) [4]. Further, people with AML often suffer a reduced quality of life with many survivors report ongoing deficits [5–8].

Some adverse effects of treatment (sometimes occurring long after induction) can be irreversible, such as anthracycline cardiotoxicity [9], infertility [10], malnutrition [11, 12], reduced physical fitness [13, 14], reduced sexual function and satisfaction [15], cognitive decline [16], and emotional distress [17–20]. Fatigue is reported irrespective of treatment status for AML and has been repeatedly shown to impair quality of life across numerous cancer types [5, 21–24] and may also be related to reduced physical and social functioning [25]. Thorough infection prophylaxis and infection management for both bacterial and fungal infections, particularly while immunocompromised, remains an important consideration for care [26, 27].

Supportive care is a domain of medicine that works to improve quality of life and reduce complications of therapy and is an integral component of AML patient management. In the last decade, there have been significant improvements in biomarkers and therapeutics in AML leading to the addition of at least 12 new therapies [28–30]; however, supportive care evidence gaps persist. For example, transfusion supportive care and infection prevention and management are pillars of supportive care in AML and were identified as a research need in 2020 by Loke et al. in the United Kingdom [31]. Yet, the evidence supporting prophylactic platelets to prevent bleeding, the optimal haemoglobin threshold to give red cells, and the best approach to reduce mortality and morbidity from infection remains elusive, particularly in patients with AML, who are less often included in the cohorts [31]. The Multinational Supportive Care in Cancer (MASCC) 2030 ambition statements [32] to improve the state of supportive care for patients highlight that evidence-based advancements in the field need coordination, collaboration, and team-based approach. With this backdrop, we sought to determine the key research priorities in the supportive care field for AML to inform our bi-national clinical trial program in AML. Our long-term goal is to encourage collaborative research efforts to address AML supportive care needs, improving outcomes through the translation of these findings into routine care [33].

## Methods

To identify and prioritise AML supportive care research questions, we undertook a three-round Delphi consensus process between February and May 2024. The ‘Delphi’ is a flexible, iterative, and transparent process that has been used extensively in healthcare to obtain consensus with a group of experts [34–36, 36]. The Delphi process tends to refer to participants as ‘panellists’ in recognition of their expertise to form a Delphi ‘panel’. The terms panellist and participants are used interchangeably.

Participants provided consent, and the research was carried out in accordance with the Declaration of Helsinki. The study was reviewed by the Monash University Human Research and Ethics Committee (#41418).

### Delphi panel recruitment

Recruitment emails were sent by both the Australasian Leukaemia and Lymphoma Group (ALLG) and investigators. The ALLG is a not-for-profit collaborative clinical trials group that sponsors, designs, and conducts clinical trials in haematological malignancies across Australia and New Zealand. The ALLG’s scientific working parties have members with expertise in their relevant areas. Specifically, members of the ALLG Supportive Care (who self-selected as having AML expertise) and AML scientific working parties were invited via email to participate in the Delphi.

The investigators invited additional clinicians (both medical and allied health) with AML expertise known to them in Australia and New Zealand. Eligibility for medical and allied health professionals included self-determined expertise in caring for people with AML with their predominant professional experience within the Australian and/or New Zealand context.

The Leukaemia Foundation (Australia) assisted with identifying and inviting people with lived experience of AML to participate in the Delphi. Lived experience included patients with a diagnosis of AML, people who have cared for someone with AML, or family members who may have also been carers or have been exposed to the burden of AML without personally identifying as a patient’s carer.

The required sample size for a Delphi is contentious with published works ranging between 10 and 1000 or more participants, but smaller sample sizes have been found to be adequate as long as participants are suitably expert in the topic [37–40]. The attrition also varies between studies and sits between 16 and 98% [35]. Therefore, to ensure we had at least 10 participants by the final round (allowing for three rounds) and accounting for a 50% attrition rate, the study aimed to enrol 40 participants, including at least 10 patient representatives, in round one.

### Study procedures and analysis

To obtain consent, the initial email invitation directed participants to an online consent process, and once received, the participant was auto-redirected to round 1 of the Delphi survey. Those providing consent were invited to all three rounds. At the beginning of each round, brief demographics of participants were collected: gender, age, experience with AML (years), location (metropolitan or rural), and discipline (a medical specialty, nursing, or allied health). People with lived experience could also select if they were a patient, carer, or family member. All data were collected online using the Qualtrics platform (Provo, Utah, USA).

#### Round 1: identifying supportive care research priorities

Round 1 asked participants to ‘*Please provide between 1 and 5 supportive care research priorities in AML you believe should be addressed *via* further research’.* Responses were collated, with duplicate and ambiguous statements removed from the analysis. A conventional content analysis was used, whereby responses were systematically coded and categorized into themes that we termed ‘domains’ to create a list of research questions that the participants considered in round 2 [41, 42]. The coding (which was inductive) was undertaken by CP (an experienced qualitative researcher), with regular meetings with ZM (a specialist haematologist and clinical researcher) to ‘sense-check’ and discuss in detail the coding and the domains. The research questions were derived to capture the participant responses and were developed through discussion amongst investigators and confirmed via participants in round 2, where changes to wording or additional questions could be suggested. Table 1 shows an example of the domain ‘transfusion supportive care’, the respective codes that created that domain, the participant responses, and the derived research questions.

**Table 1:** Domain of ‘transfusion supportive care’ with excerpt participant responses, coding, and the derived research question

Coding (related to this domain)	Participant responses	Derived research questions
Transfusion support	*‘Transfusion support’*	*
	*‘Guidelines and parameters for transfusion support’*	
	*‘Transfusion support—target thresholds for red cells and platelets not well defined or evidence based’*	
Transfusion thresholds	*Transfusion thresholds (haemoglobin and platelet counts), with a particular focus on outpatients with chronic disease and/or therapy-induced cytopenias (the latter being a growing group)*	What is the optimal haemoglobin threshold for red cell transfusion support? What is the optimal platelet count threshold for platelet transfusion? Can quality of life be used to guide red cell transfusion support?
	*Optimal threshold for red cell transfusion support*	
	*‘Transfusion support—target thresholds for red cells and platelets not well defined or evidence based’*	
	*‘Quality of life guided thresholds’*	
Haemostasis	*‘Interventions to prevent bleeding in patients with severe thrombocytopenia’*	Which interventions are more effective to prevent bleeding in patients with severe thrombocytopenia?
	*‘Transfusion research eg. platelet transfusions in the prophylaxis setting’*	
Managing risks	*‘Reducing the long term impacts of ongoing transfusions…’*	How can the long-term impacts of transfusion be minimised?
	*‘Transfusion research eg. platelet transfusions in the prophylaxis setting’*	
Transfusion guidelines	*‘Guidelines and parameters for transfusion support’*	What evidence is needed to improve guidelines for transfusion support?
	*‘Transfusion support—target thresholds for red cells and platelets not well defined or evidence based’*	

*Participant responses in this code were captured within other derived questions (see transfusion guidelines and transfusion threshold codes)

#### Round 2: rating each research question

Opening in April 2024, round 2 asked participants to indicate each research question’s level of importance on a 4-point Likert scale (where 1 was ‘very important/urgent priority’ and 4 was ‘not important/not a priority’). A terminology sheet was developed to assist non-clinical participants with medical terminology. There was a provision in the survey to suggest alternative wording to the research questions posed, as well as an opportunity to suggest any research questions that had been missed. Participants could download a copy of their own responses to refer to in round 3.

A mean and standard deviation were calculated for each research question, which was used to review whether the research question reached a minimum consensus criteria determined a priori(although there are no agreed consensus criteria in Delphi studies [43]). The consensus was defined as 70% or more participants providing a positive result (Likert score 1 or 2), and the mean score was < 2. Research questions meeting the consensus criteria went through to round 3 for consideration.

#### Round 3: ranking research questions for an order of priority

In the final round, participants were asked to rank their top 10 research questions in order of priority. Additionally, for each research question, participants selected what type of research was most urgently needed to address the question from a choice of; aetiological/biological, exploratory, intervention development, randomised controlled trial, or implementation research.

To combine responses from all participants and obtain the order of priority of the research questions, the ranking was reverse coded where a ranking of 1 was allocated 10 points, a ranking of 2 was allocated 9 points, and so on. Rankings > 10 were excluded which occurred when participants rated beyond 10 research questions. Scores were summed to generate a prioritised list of supportive care research questions for AML, where the highest score was ranked first. Where scores were the same, a median score was used to determine the rank priority.

## Findings

### Demographics

Table 2 describes participant demographics across each of the Delphi rounds. Most participants were female, were aged between 41 and 60 years of age, had more than 6 years of experience with AML, and lived or worked in a metropolitan area. Rounds one and two had only Australian participants, while round three included two panellists contributing from New Zealand. Participating medical professionals included haematologists, infectious disease physicians, and specialist nurses, including nurse practitioners. A further breakdown is not provided due to the re-identifiable nature of low participants in each group. Approximately one-third of respondents had lived experience of AML and all identified as patients.

**Table 2:** Demographics across Delphi rounds

	Round 1	Round 2	Round 3
**Participants (total)**	31	23	22
**Gender**	*N* (%)	*N* (%)	*N* (%)
Male	6 (19)	5 (22)	6 (27)
Female	25 (81)	18 (78)	15 (68)
Third gender	-	-	1 (5)
**Age group (years)**	*N* (%)	*N* (%)	*N* (%)
18–40	10 (32)	7 (30)	6 (27)
41–60	16 (52)	11 (48)	13 (59)
≥ 61	5 (16)	5 (22)	3 (14)
**Location**	*N* (%)	*N* (%)	*N* (%)
Metropolitan area	23 (74)	14 (61)	15 (68)
Regional/rural area	8 (26)	9 (39)	7 (32)
**Role and experience with AML**	*N* (%)	*N* (%)	*N* (%)
**Medical (physicians and nursing)**	19 (61)	11 (48)	15 (68)
Experience with AML (years)			
≤ 5	4 (21)	1 (9)	2 (13)
6–10	5 (26)	3 (27)	4 (27)
≥ 11	10 (53)	7 (64)	9 (60)
Other professionals*	2 (6)	2 (9)	1 (5)
Experience with AML (years)			
≤ 5	-	-	
6–10	2 (100)	1 (50)	1 (100)
≥ 11	-	1 (50)	
**People with lived experience**	10 (33)	10 (43)	6 (27)
Experience with AML (years)			
≤ 5	3 (30)	4 (40)	3 (50)
6–10	5 (50)	3 (30)	2 (33)
≥ 11	2 (20)	3 (30)	1 (17)

*Other professionals included allied health, research professionals or scientists

### Round 1

Round 1 had 31 participants, which through the content analysis, elicited 68 research questions across 13 domains: transfusion support, infection prevention and management, microbiome research, nutrition support, exercise and physical functioning, psychosocial support, symptoms, side effects, survivorship and late effects, frailty, fertility preservation, pain management and palliative care, access to therapies, complementary and alternative medicines, and models of care (supplementary material S1). The largest domain was psycho-social supportive care, with 12 research questions, whilst the smallest domains were ‘frailty’ and ‘access to therapies’, with only one research question each.

### Round 2

Twenty-three of the original 31 respondents participated in round 3 (response rate 74%) and rated the research questions identified in round 1. Sixteen research questions met the a priori consensus criteria (Table 3) to go through to round 3. None of the research questions met the minimum criteria for consensus in the domains of; microbiome, psycho-social supportive care, access to therapies, and complementary and alternative medicines. In round 2, there was revised phrasing for one question from: ‘How can criteria be established for better integration of palliative care at an earlier or optimal time point?’, to, ‘What criteria should be established for an optimal time point for better integration of palliative care?’. Participants raised no additional research questions.

**Table 3:** Mean and standard deviation of the rating for each research question

Domain and research question	Mean (SD)	Panellists responding 1 or 2 (%)
Transfusion supportive care		
Which interventions are more effective to prevent bleeding in patients with severe thrombocytopenia?	1.64 (0.73)	86
What is the optimal platelet count threshold for platelet transfusion?	1.73 (0.70)	86
Infection prevention and management		
What are the benefits and harms of prophylactic antibiotics in people with AML?	1.57 (0.75)	86
What is the most effective regime to treat recurrent episodes of culture negative febrile neutropenia?	1.62 (0.59)	95
Are long or short courses of antibiotics more effective in febrile neutropenia?	1.81 (0.60)	90
Nutrition		
How can nutrition support be optimised to support recovery from deconditioning?	1.77 (0.69)	86
Exercise and physical conditioning		
What is the effectiveness of an exercise intervention in improving survival, health, and psychosocial outcomes for people with AML?	1.68 (0.72)	86
Symptoms, side-effects, survivorship, and late-effects		
How can treatment regimens be optimised to reduce disease and medication side-effects?	1.77 (0.53)	96
What interventions are effective at managing various symptoms and side-effects?	1.86 (0.64)	86
Frailty		
Can a frailty assessment guide risk stratification and decision-making (e.g. intensive vs. non-intensive therapy)?	1.73 (0.70)	86
Fertility preservation		
What are the best strategies for fertility preservation, particularly in younger women having intensive and allogeneic transplantation?	1.59 (0.67)	91
Pain management and palliative care		
How can criteria be established for better integration of palliative care at an earlier or optimal time point?	1.67 (0.86)	86
Models of care		
How does the frequency of outpatient monitoring during outpatient delivery of chemotherapy (e.g. 2 times per week vs. 3 times per week) affect quality of life, patient outcomes, and costs of care?	1.91 (0.75)	86
What models of care are most effective (including costs) at reducing hospitalisations?	1.64 (0.66)	91
What models of care are most effective at reducing infections?	1.55 (0.67)	91
How can models of care be improved to enable patients to spend more time at home, e.g. a contactable person available out of hours who has AML-specific knowledge?	1.55 (0.60)	96

### Round 3

Seventy-one percent (response rate of 71%) of the original panel of experts determined that the most important supportive research question to be addressed was ‘What are the benefits and harms of prophylactic antibiotics in people with AML?’ (Table 4) and that randomised controlled trials (RCT) should be undertaken to address this question. RCTs were thought to be needed to address 4 out of the 10 research questions, while intervention development and exploratory research were needed for the remaining questions. The domain most represented in the final rankings was ‘infection prevention and management’ (3 research questions), followed by ‘transfusion supportive care’ (2 research questions).

**Table 4:** Top 10 supportive care research questions for AML

Rank	Domain	Research question	Type of research to address the research question	Round 2 rating (mean)
1	Infection prevention and management	What are the benefits and harms of prophylactic antibiotics in people with AML?	RCT	1.57
2	Transfusion supportive care	Which interventions are more effective to prevent bleeding in patients with severe thrombocytopenia?	RCT	1.64
3	Infection prevention and management	Are long or short courses of antibiotics more effective in febrile neutropenia?	RCT	1.81
4	Frailty	Can a frailty assessment guide risk stratification and decision-making? (e.g. intensive vs. non-intensive therapy)	Intervention development	1.73
5	Nutrition	How can nutrition support be optimised to support recovery from deconditioning?	Exploratory	1.77
6	Infection prevention and management	What is the most effective regime to treat recurrent episodes of culture-negative febrile neutropenia?	Intervention development	1.62
7	Exercise and physical conditioning	What is the effectiveness of an exercise intervention in improving survival, health, and psychosocial outcomes for people with AML?	Intervention development	1.68
8	Transfusion supportive care	What is the optimal platelet count threshold for platelet transfusion?	RCT	1.73
9	Models of care	What models of care are most effective at reducing infections?	Exploratory	1.55
10	Pain management and palliative care	How can criteria be established for better integration of palliative care at an earlier or optimal time point?	Exploratory	1.67

### Open-ended responses

Seven panellists provided responses to the open-ended question in round 2. Two people with lived experience commented about the complexity of the questions, specifically that the terminology sheet was helpful and that the process illuminated the complexity of care and the difficulty in prioritising one aspect over another. Two panellists provided suggestions that were already captured within the research questions presented. Another two people emphasised the issues of resourcing and implementation even if an intervention was shown to be beneficial. One panellist took issue with the methodology and felt that proposed actions rather than research questions would have been more suitable.

## Discussion

This Delphi consensus study is the first such study to identify a ‘top 10’ supportive care research questions to address some of the most pressing supportive care needs for people with AML in Australia and New Zealand. Using an online methodology allowed us to include geographically diverse participants and also allowed for participant anonymity, where individuals were unable to influence others with their opinion [36]. Another key strength of this study was that the panel included people with lived experience, therefore capturing views and experiences in research priority setting that are often missed. Our consensus study was limited by the expertise of those on the panel. For example, some allied health specialties (e.g. nutrition) were absent despite our efforts to recruit persons with such expertise. Nonetheless, a nutrition research question was in the top 10 list, perhaps suggesting a recognition of our panel members, of a multi-speciality appreciation and approach to AML care.

Three of the top 10 research questions concerned infection prevention and management, particularly the role of prophylactic antibiotics, course length of antibiotics for febrile neutropenia, and management of recurrent episodes of culture-negative febrile neutropenia. Infection is an important cause of morbidity and mortality in patients with AML [44]. There have been previous trials of different infection prevention strategies in patients with neutropenia and AML, yet conflicting results with respect to their effectiveness, and these strategies also need to take into consideration concerns regarding antibiotic resistance [45]. These issues are made more complex with an environment of increasing antimicrobial resistance, evolving immune-targeted cancer therapeutics and consequential drug-drug interactions. Additionally, it remains unclear whether infection risk can be attributed to the novel therapies or due to underlying disease or other patient characteristics [27]. While not documented in our study, antifungal prophylaxis presents similar challenges [46].

The second most frequent domain in the top 10 list in this study was improving evidence around transfusion support. Evidence is limited and there have been few studies to inform the current practice, even though blood components remain a critical component of supportive care [47, 48]. For example, there have been studies assessing the use and dosage of platelet prophylaxis to prevent bleeding, such as the TOPPS [47] and PLADO [48] trials, yet bleeding still occurred in these patients. Bleeding therefore remains to be an ongoing concern for clinicians and their patients.

Palliative care research additionally featured in our top 10, perhaps in the recognition of the unique and differing needs of haematological malignancies compared with solid organ tumours and highlighting research needed to better support patients with haematological malignancies at end of life [49, 50].

Despite psychosocial care research questions dominating round 1 of our study, none of the proposed research questions reached the minimum consensus criteria. In contrast to our study, other supportive care research prioritisation literature in cancer, more generally, tends to rank research into psychological support highly [51, 52]. This difference may be due to a lack of familiarity of the issues by respondents or an already extensive body of literature demonstrating the benefits of psycho-social care in oncology [53, 54, 54–57]. Even though such evidence exists, and as many as 30% of cancer patients report psychosocial distress and mental disorders [58], many patients find it difficult to access specialist psychosocial services due to inadequate provision of resources, poor integration of services that are in place, fiscal restraints within models of care, and inadequate numbers of professionals in the field [57, 59–62].

Other efforts to prioritise supportive care research, specifically in AML, are sparse. Loke et al., in a recent commentary, highlighted the need for improved evidence for transfusion practices and infection management in people with AML, which aligned with priorities 1 and 2 in the present study [31]. In mid-2024, The Multinational Supportive Care in Cancer (an internationally renowned professional organisation devoted to supportive care in cancer) released an overarching framework to guide supportive care development globally: Supportive Care 2030 Movement ambition statements [32]. These unifying statements discuss topics such as ‘guideline development and implementation, coordinated and individualised care, dedicated supportive oncology services, self-management, needs for screening and actions, patient education, behavioural support, financial impact minimisation, comprehensive survivorship care, and timely palliative care’. Whilst specific topics such as palliative care overlap with the present study’s findings, importantly, the ambition statements highlight the need for collaboration, coordination, and team-based approaches across all levels. Highly collaborative novel methodologies, such as the use of platform trials, have the potential to expedite findings as they provide an ideal opportunity to prospectively assess multiple supportive care measures in one study. Perhaps the growing use of co-operative group platform trials to explore molecularly-targeted therapies in AML [63] could consider supportive care research arms alongside novel therapeutics, either within all trial domains or only for participants who are not eligible for allocation to a molecularly targeted therapy or whom are too unfit or frail for therapeutic arms.

Working collaboratively and making the best use of novel trial approaches will be paramount to achieving any progress towards improving the evidence for supportive care in AML.

## Conclusions

Improving supportive care for people with AML can only be achieved through collaborative efforts to undertake high-quality research. The development of a top 10 list of prioritised research questions provides a research roadmap for clinicians and researchers to inform future clinical supportive care research activities.

## Supplementary Information

Below is the link to the electronic supplementary material.

Supplementary file 1 (DOCX 24.2 KB)

## Data Availability

The data that support the findings of this study are available upon reasonable request and pending approval by the owning ethics committee.
