# Supplementary material for: Research priorities to address supportive care needs in acute myeloid leukaemia (AML)—results from a Delphi survey
Source: Support Care Cancer. 2025 Sep 11;33(10):844. doi: 10.1007/s00520-025-09888-7 (PMC12426066; doi:10.1007/s00520-025-09888-7)
Supplement: Supplementary file 1 — (DOCX 24.2 KB) [file 520_2025_9888_MOESM1_ESM.docx]

S1. AML Supportive Care Research Questions: results from the round 1 content analysis.

| **Domain** | **AML Supportive Care Research Questions from Round 1** |
| --- | --- |
| Transfusion supportive care | What evidence is needed to improve guidelines for transfusion support? |
|  | Can quality of life be used to guide red cell transfusion support? |
|  | What is the optimal haemoglobin threshold for red cell transfusion support? |
|  | What is the effectiveness of prophylactic platelet transfusions to prevent bleeding complications? |
|  | Which interventions are more effective to prevent bleeding in patients with severe thrombocytopenia? |
|  | What is the optimal platelet count threshold for platelet transfusion? |
|  | How can the long-term impacts of transfusion be minimised? |
| Infection prevention and management | What is the optimal prophylaxis approach for antifungal therapies? |
|  | Which patients treated with lower-intensity therapies (e.g. venetoclax and azacitidine) should receive prophylactic mold-active azoles? |
|  | What are the benefits and harms of prophylactic antibiotics in people with AML? |
|  | Can the net state of immunosuppression be used to predict prevention risk and therefore guide prophylaxis? |
|  | What is the most effective regime to treat recurrent episodes of culture negative febrile neutropenia? |
|  | Are long or short courses of antibiotics more effective in febrile neutropenia? |
|  | What is the role of ambulatory monitoring devices for early detection of, and early intervention for, febrile neutropenia? |
|  | Should GCSF therapy be routinely used during induction with 7+3 and/or venetoclax-based regimens? |
| Microbiome | What interventions are most effective for bowel recovery following intensive chemotherapy (eg faecal microbiotal transplant or capsule formulations)? |
|  | What interventions are most effective for bowel recovery following parenteral antibiotics (eg faecal microbiotal transplant or capsule formulations)? |
|  | What is the role of gut and lung microbiome in treatment and recovery? |
| Nutrition | How can nutrition support be optimised to support recovery from deconditioning? |
|  | What is the role for nutrition support in patients undergoing exercise interventions? |
| Exercise and physical conditioning | What is the optimal exercise 'prescription' including the exercise type, duration and mode of delivery? |
|  | What is the effectiveness of an exercise intervention in improving survival, health and psychosocial outcomes for people with AML? |
|  | What are the long-term outcomes of exercise interventions? |
|  | How can adherence to exercise prescriptions be improved? |
|  | How can exercise interventions be provided equitably? |
|  | How can patients be supported to self-manage physical rehabilitation? |
| Psycho-social supportive care | What is the psychological impact of side-effects and late-effects? |
|  | How can those most at risk for poor psychosocial outcomes be identified and does an intervention affect survival? |
|  | How can the efficacy of psychosocial care be optimised for people with AML from diagnosis through to survivorship? |
|  | How does the hospital environment affect psychosocial outcomes in people with AML? |
|  | Are peer-support mentoring or mindfulness programs beneficial for psychosocial functioning for AML? |
|  | Which treatments and side-effects have the greatest impact on psychosocial functioning? |
|  | What information or resources do patients, families/carers need at diagnosis about possible psychosocial impacts of their care? |
|  | What information do patients want or need at different points in their journey? |
|  | What are the psychosocial needs of families and/or carers? |
|  | How can patients be empowered to self-manage psychosocial care? |
|  | How can patients be supported to have difficult conversations with family (including children) and friends? |
|  | How can patients be supported in the transition back to 'normal' life after treatment? (e.g. return to work, managing ongoing side effects etc.) |
| Symptoms, Side effects, survivorship and late-effects | How can patients be better informed and what do they want to know about possible symptoms and side-effects, particularly those who have significant or long-lasting effects ? |
|  | What information do patients want about possible late-effects before deciding on a procedure? |
|  | What are the unmet needs of patients and families regarding symptoms and side-effects? |
|  | How can treatment regimens be optimised to reduce disease and medication side-effects? |
|  | What interventions are effective at managing various symptoms and side-effects? |
|  | Is there a role for early administration of cardiac medications (e.g. A2 antagonists, ACEIs etc) to reduce the risk of late cardiotoxicities following anthracycline-based therapies? |
|  | What are the established programs (including frequency of visits) for follow-up upon completion of treatment? |
|  | What does optimal survivorship care look like? |
|  | What is the role of GPs in managing side-effects, late-effects and survivorship care? |
|  | How can patients be better identified for central venous access and monitored to prevent complications? |
| Frailty | How can current frailty measures be optimised for use in AML? |
|  | Can a frailty assessment guide risk stratification and decision-making? (e.g. intensive vs non-intensive therapy) |
| Fertility preservation | What are the best strategies for fertility preservation, particularly in younger women having intensive and allogeneic transplantation? |
| Pain management and palliative care | How can current pain management strategies be improved? |
|  | How can criteria be established for better integration of palliative care at an earlier or optimal time point? |
|  | What is the experience of patients and families with the involvement of earlier palliative care compared with those who don’t have receive it? |
| Access to therapies | How can we improve equitable access to supportive therapies (e.g. exercise physiotherapy), particularly in regional or rural areas? |
| Complementary and alternative medicines (CAMs) | How many AML patients are using CAMs, what CAMs are they using and how often? |
|  | What concerns are not being addressed by health services that patients are using CAMs to address? |
|  | What are the harms and benefits of CAMs for AML? |
| Models of care | How long should patients receiving cycle 1 ven-aza be in hospital? |
|  | How safe is hospital discharge after the completion of chemotherapy ? |
|  | How does the frequency of outpatient monitoring during outpatient delivery of chemotherapy (e.g 2 times per week vs 3 times per week) affect quality of life, patient outcomes and costs of care? |
|  | How should outpatient chemotherapy be delivered and what patients are suitable for outpatient chemotherapy? |
|  | What models of care are most effective (including costs) at reducing hospitalisations? |
|  | What models of care are most effective at reducing infections? |
|  | How can models of care be improved to enable patients to spend more time at home e.g. a contactable person available out of hours who has AML-specific knowledge? |
